# Direct aperture optimization using an inverse form of back‐projection

**DOI:** 10.1120/jacmp.v15i2.4545

**Published:** 2014-03-06

**Authors:** Xiaofeng Zhu, Timothy Cullip, Gregg Tracton, Xiaoli Tang, Jun Lian, John Dooley, Sha X Chang

**Affiliations:** ^1^ Department of Radiation Oncology University of North Carolina at Chapel Hill Chapel Hill NC USA

**Keywords:** IMRT, DAO, MART, VMAT index

## Abstract

Direct aperture optimization (DAO) has been used to produce high dosimetric quality intensity‐modulated radiotherapy (IMRT) treatment plans with fast treatment delivery by directly modeling the multileaf collimator segment shapes and weights. To improve plan quality and reduce treatment time for our in‐house treatment planning system, we implemented a new DAO approach without using a global objective function (GFO). An index concept is introduced as an inverse form of back‐projection used in the CT multiplicative algebraic reconstruction technique (MART). The index, introduced for IMRT optimization in this work, is analogous to the multiplicand in MART. The index is defined as the ratio of the optima over the current. It is assigned to each voxel and beamlet to optimize the fluence map. The indices for beamlets and segments are used to optimize multileaf collimator (MLC) segment shapes and segment weights, respectively. Preliminary data show that without sacrificing dosimetric quality, the implementation of the DAO reduced average IMRT treatment time from 13 min to 8 min for the prostate, and from 15 min to 9 min for the head and neck using our in‐house treatment planning system PlanUNC. The DAO approach has also shown promise in optimizing rotational IMRT with burst mode in a head and neck test case.

PACS number: 87.55.D‐

## I. INTRODUCTION

The goal of optimizing intensity‐modulated radiation therapy (IMRT) is to maximize tumor killing while minimizing its toxicity to neighboring organs at risk (OAR) for each individual patient. The conventional optimization is a two‐step process starting with optimizing pencil beam‐based intensity maps, also termed as fluence map optimization. The next step is to convert the optimized fluence maps into a number of segment fields formed by multileaf collimators (MLC). Algorithms for IMRT leaf sequencing have been developed and improved to reduce total segment numbers in order to shorten treatment delivery time.^1^, ^2^, ^3^, ^4^ Recent studies indicate that shortening treatment time could reduce dose delivery uncertainty due to organ motion and patient setup for image‐guided radiotherapy, adaptive radiotherapy, and radiotherapy quality assurance.^5^, ^6^, ^7^, ^8^ In addition to dosimetric quality, the workflow efficiency of the cancer clinic is also affected by long treatment time. We need to find new approaches to improve the quality and efficiency of state‐of‐the‐art radiotherapy.

Direct aperture optimization (DAO), which directly models the complex segment shapes and their corresponding beam‐on time, was first adopted for step‐and‐shot IMRT and solved with a simulated annealing optimization scheme.^(9)^ After applying speed constraints of gantry rotation and MLC leaf motion, DAO provided a robust solution for intensity‐modulated arc therapy.^(10)^ Utilizing the freedom of linac gantry rotation, arc therapy features much shorter treatment times than static IMRT, and single‐arc delivery was achieved by integrating progressive sampling during optimization.^(11)^ In additional to those stochastic approaches, direct machine parameter optimization (DMPO) starts with a pseudo‐optimized plan with a predefined number of expected segments. MLC leaf positions and segment weights are further optimized using a gradient search. The gradient search uses a global objective function (GOF), which is calculated by an opening density matrix.^12^, ^13^, ^14^ Another approach for DAO is a polynomial time algorithm using column generation. This approach selects an optimized MLC shape from thousands of feasible combinations, and the concept of ‘price’ in the Simplex method is applied for each pencil beam, and the Karush‐Kuhn‐Tucker condition is used to verify whether a solution is optimal.^(15)^ Powered by GPU parallel computing, dose optimization of volumetric‐modulated arc therapy (VMAT) can be solved in minutes following the column generation approach.^16^, ^17^ All the aftermentioned DAO methods are built upon a GOF that can be expensive to compute and optimize, especially when the noncovex feature of dose‐volume histogram (DVH) constraints needs to be included.^18^, ^19^

We propose a different DAO method which does not use a GOF. Our method utilizes an inverse form of back‐projection used in CT reconstruction. The first CT reconstruction in history was accomplished by iteratively reducing the deviations of the predicted detector signal from the measured signal, using the algebraic reconstruction technique.^20^, ^21^ In IMRT optimization, we search for a configuration of beam intensities that deliver the prescribed dosimetry. The “unknown” and “known” in radiotherapy optimization are reversed compared to those in CT reconstruction: the signal of CT detector (“known” in CT) is replaced by the intensity of Y ray pencil beam (“unknown” in IMRT); the expected dose at central target (“known” in IMRT) replaces the electron density to be reconstructed (“unknown” in CT). Similar image reconstruction methods have been proposed and applied to conformal radiotherapy,^22^, ^23^, ^24^ and recently been applied to improve VMAT optimization.^(25)^ In this work, multiplicative algebraic reconstruction technique (MART) in CT reconstruction is modified as a simple solution for DAO in our in‐house treatment planning platform.

Our approach is based on the index concept. The index, equivalent to the multiplicand in MART, is defined as the ratio of the targeted dose over the current computed dose. The optimization process is intended to drive the index value to ‘1’ for all the voxels, including planning target volume (PTV) and organ at risk (OAR). Not all the index could reach ‘1’ after optimization, as the prescribed dose distribution may not be physically possible. The tradeoffs of voxel index optimization are used, and this is controlled by the weight assigned for each voxel in the optimization. A large weight is more likely to drive its index to reach ‘1’, and reach its optimized dose value. Our group has developed a back‐projection and index‐based gradient optimization approach, where fluence map optimization is achieved by assigning a index for each beamlet.^(26)^ We have implemented this approach for clinical use since 1996. In this work, the index concept is extended to optimize MLC segment shape and segment weight, and to create new segments to achieve the goal of DAO for treatment plans of good quality and fast delivery.

## II. MATERIALS AND METHODS

Index‐based DAO is implemented in C/C++ in our in‐house treatment planning platform, PLanUNC (Department of Radiation Oncology, University of North Carolina, Chapel Hill, North Carolina). We integrated model guided rendering (MGR) into PlanUNC to visualize the optimization results. MGR utilizes partial image segmentations as a framework for combining information from multiple data sources into a single view.^(27)^ Since 2011, our DAO approach has been used clinically for step‐and‐shoot IMRT patient treatment. DAO for arc therapy is also under development. Using rotational IMRT with burst mode, a head and neck case was retrospectively studied.

**Figure 1:**
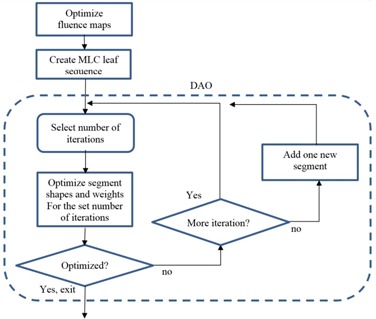
The flowchart shows our interactive planning process. The first two steps provide a starting condition for DAO enclosed by the dashed frame. After MLC leaf sequencing, a planner defines the number of iterations to optimize the segment shapes and segment weights. The planner can add a new segment between any set of iterations.

**Figure 2:**
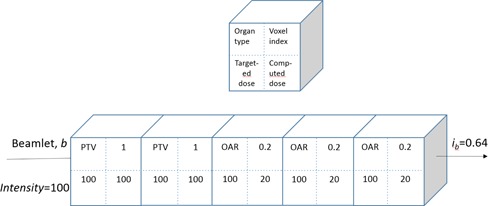
Illustration of ray tracing of the beamlet. The top insert indicates the definitions of the four parameters inside each voxel: organ type (top left), computed dose (bottom left), targeted dose (bottom right), and index (top right). Voxel index is defined as the ratio of targeted dose value over the current dose value. The targeted doses for the oar and PTV are 20 and 100 units, respectively. The beamlet with an intensity = 100 deposits 100 unit of dose at each voxel. For simplification, the weight factors are set to 1 for all five voxels. The beamlet index is the average of the weighted indices, $i_{b}=0.64$. As an attempt to approach 1 for beamlet index, the beamlet intensity will be multiplied by the index, and thus, reduced to 64 units by the end of this iteration.

Figure 1 shows a flowchart of our DAO approach, where DAO starts after fluence map optimization and MLC leaf sequencing. For fluence map optimization, each beam is divided into hundreds of beamlets. A beamlet is a single pencil beam for ray tracing with a spatial resolution of a few millimeters along the beam path. Beamlet intensities are iteratively optimized using the index for each voxel, as illustrated in Fig. 2 and discussed in the Materials and Methods section A below. Then, leaf sequencing proceeds with a coarse sampling (e.g., 10 mm in spatial resolution and three levels in beamlet intensity stratification). Such coarse sampling often generates a plan with fewer segments, but degraded quality compared with, for instance, leaf sequencing sampled with 3 mm and eight levels. Working upon this pseudo‐optimized plan with preexisting MLC segments, DAO is implemented interactively. Unlike DMPO, we do not specify the total number of segments. Instead, the planner specifies the number of iterations. Each iteration takes a few seconds and, often, the optimization will converge within ten iterations. If further optimization is needed, the user may request more iterations, modify optimization objective parameters, or add a new segment whose segment shape is determined by the cost computed using the indices, then run more iterations. Once the number of segments for one beam reaches ten, adding more segments into this beam rarely improves the result.

**Figure 3:**
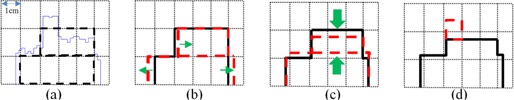
One‐dimensional beamlet profile is approximated by one leaf pair. The dotted grids show the sampling resolution. The difference (a) after leaf sequencing is shown between the expected profile (solid line) and that from two MLC segments (dash line); (b) solid line shows current MLC segments, and dash lines show leaf position adjusted using beamlet index near the leaf edge; (c) dashed line shows adjusted segment weight using segment index; (d) new segment (dash line) is added using the column generation method.

Figure 3 shows a simplified one‐dimensional example for DAO. The vertical axis shows the beamlet intensity. Vertically, three levels are selected to stratify the intensities. The horizontal axis shows the beamlet position and the MLC leaf position. Horizontally, a step size of 10 mm for MLC was chosen to sample the beamlet intensity profile. In Fig. 3(a), a beamlet has a spatial resolution of 1.3 mm (solid line). After leaf sequencing, two MLC segments are generated to approximate the beamlet profile. Those two segments are plotted with dash lines in Fig. 3(a), with one segment laid on top of the other. To reduce difference between the original profile and the approximation, three methods are discussed: optimizing segment shapes (Fig. 3(b) and section B below), optimizing segment weights (Fig. 3(c) and section C below), and adding new segments (Fig. 3(d) and section D below).

### A. Index

The index indicates how much the current dose should be increased or decreased. Each voxel is assigned an index. To optimize the fluence map, a beamlet index is introduced by normalizing the weighted voxel index along the beam. The beamlet intensity is tuned by multiplying it by its beamlet index. Figure 2 illustrates one iteration of a beamlet intensity optimization. During DAO, the beamlet index is also used to optimize segment shape. Moreover, to optimize segment beam‐on time, a segment index is introduced by normalizing the beamlet index within the segment.

For each voxel, its index varies according to the organ type the voxel belongs to. For instance, the weighted index for a voxel v inside PTV is formulated as:

(1) $$i_{v}=\omega_{v}\frac{{\text{dose}}_{v}^{p}}{{\text{dose}}_{v}}$$

$\omega_{i}$ defines this voxel's importance or weight factor during optimization, similar to the weight parameters used in the optimizer of commercial treatment planning systems; $dose_{v}^{p}$ is the prescription (targeted) dose, and $dose_{v}$ is the current dose to be optimized. For a voxel inside OAR, if $dose_{v}^{p}$ is the maximum allowed dose, and its weighted index is

(2) $$i_{v}=\{\begin{array}{ll} {\left(\frac{{\text{dose}}_{v}^{p}}{{\text{dose}}_{v}}\right)}^{\omega_{v}}, & {\text{dose}}_{v}>{\text{dose}}_{v}^{p}\\ 1, & {\text{dose}}_{v}\leq {\text{dose}}_{v}^{p} \end{array}$$

$i_{v}=1$ indicates that no change will be applied since current dose is less than allowed, and the sparing goal for the OAR is achieved. Figure 2 gives an example of the index calculation and resulting beamlet intensity change. For simplification, weight factors (not shown in Fig. 2) are set to 1 for all five voxels.

Finally, each beamlet is assigned an index by normalizing the weighted index of all the voxels along the beamlet track:

(3) $$I_{b}=\frac{\sum_{v\in b}i_{v}}{\sum_{v\in b}\omega_{v}}$$

Beamlet index provides an effective and efficient solution for IMRT fluence map optimization.^(26)^

**Figure 4:**
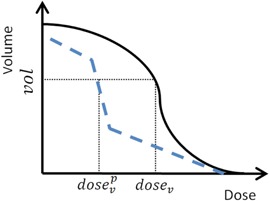
For DVH constraints, index at voxel v equals $dose_{v}/dose_{v}^{p}$. The dashed line is the goal DVH. The solid line is the current DVH. For any nominal dose, $dose_{v}$, its index is computed as $dose_{v}^{p}/dose_{v}$. The nominal volume is interpolated using $dose_{v}$ from the current DVH curve. The nominal $dose_{v}^{p}$ is interpolated using volume from the goal DVH curve.

For DVH constraints, we do not use the GOF approach introduced by Wu and Mohan^(28)^ and Carlsson.^(14)^ The DVH constraints are applied using the index, comparing the goal DVH and current DVH for each nominal dose. In Fig. 4, at the start of each iteration, the current DVH (solid curve) will be updated. For any nominal dose value, $dose_{v}$, a volume is interpolated from current DVH curve. Then, the volume is used to interpolate $dose_{v}^{p}$ from the goal DVH (dashed curve). A ratio is computed as $dose_{v}^{p}/dose_{v}$ for every $dose_{v}$. As the beamlet is passing through each voxel during the ray‐tracing process, the index for each voxel will be set to the computed ratio.

### B. optimizing segment shapes using beamlet index

For beamlets located near a segment edge, $I_{b}$ indicates where the leaf position should be moved. If the beamlet hits the leaf, the leaf is closed to that beamlet, otherwise the leaf is open to that beamlet. If the leaf is open, the beamlet intensity is on; otherwise, its intensity is off. The resulting beamlet index will increase or decrease, based on the ratio of the current beamlet intensity versus that desired. An empirical method of optimizing segment shapes is implemented by checking the index of the beamlet. The shape adjustments are shown by the arrows in Fig. 3(b). During each iteration, the leaf opening will be increased by 1 mm if the beamlet passing through the segment edge has its index $I_{b}>1.007$, as an indication of under dose. The opening will be decreased by 1 mm if it is over dose, as indicated by $I_{b}<0.992$.

A heuristic method is used to accelerate segment shape optimization. The aforementioned 1 mm leaf position adjustment is small, and five iterations may be needed to search a range of 5 mm. For instance, one approach in arc therapy is to convert a number of static beams (24 or 36 beams) into a continuous arc plan. A segment will move to a new gantry position, then change to a new shape. This may require a larger than 1 mm position change of the MLC leaves.^13^, ^29^, ^30^ The index is used to optimize the shape by searching within a neighboring range defined by the planner, 5 mm for example. At a segment s, its beam on time is denoted as $w_{s}$. Thus, the pencil beamlet intensity is either $w_{s}$ or zero. We assign $dose_{v}^{on}$ and $dose_{v}^{off}$ as the resulting doses with beamlet on and off, and $i_{v}^{on}$ and $i_{v}^{off}$ as the beamlet index with beamlet on and off, respectively. Here, a local objective function for each beamlet could be formulated as:

(4) $${\text{obj}}_{b}=\sum_{v\in b}\omega_{v}({\text{dose}}_{v}-{{\text{dose}}_{v}^{p})}^{2}$$

Using the beamlet index, a variation of $obj_{b}$ when the beamlet is set ‘on’ or ‘off’ is formulated as:

(5) $$\begin{array}{lll} \Delta obj_{b} & = & \sum_{v\in b}\omega_{v}[{\left({\text{dose}}_{v}^{\text{on}}-{\text{dose}}_{v}^{p}\right)}^{2}-{\left({\text{dose}}_{v}^{\text{of}f}-{\text{dose}}_{v}^{p}\right)}^{2}]\\ & = & \sum_{v\in b}\omega_{v}{\left({\text{dose}}_{v}^{p}\right)}^{2}\left[{\left(i_{v}^{\text{on}}-1\right)}^{2}-{\left(i_{v}^{\text{of}f}-1\right)}^{2}\right] \end{array}$$

To minimize the objective function value, a contiguous set of ‘on’ or ‘off’ beamlets is chosen where the sum of $\Delta obj_{b}$ is the most negative. Using the current segment weight $w_{s}$, once the beamlets' intensities are chosen, new leaf positions are derived as the edges enclosing the contiguous beamlets which are ‘on’.

### C. Optimizing segment weights using segment index

With the segment shapes fixed, segment weights are optimized using the index for each segment. The index of the segment indicates the ratio of the beam on time versus that required to supply the targeted dose. The photon flux passing through the segment opening is viewed as a cone‐beam. For a segment s, imagine the cone beam is divided into N pencil beamlets, each with its index $I_{b}$. The index for the cone beam is defined as:

(6) $$C_{s}=\sum \frac{I_{b}}{N}$$

After the mth iteration, the weight of the segment s is updated:

(7) $$w_{s}^{m+1}=w_{s}^{m}*C_{s}$$

The arrows in Fig. 3(c) depict the changes in the segment weights accordingly. Compared with segment shape optimization, weight optimizing is simpler as the number of variables is smaller. Using a GOF, weight optimization can also be solved by linear model, conjugated gradient or quasi‐newton methods.

### D. Adding a new segment using index

As shown in Fig. 3(d), the user can add a new segment shape to better approximate the original beam profile. Since there are millions of possible MLC shapes, a column generation technique is the most efficient method known for solving similar problems. Instead of generating all possible shapes at start, a new shape is produced only if needed. The cost of a beamlet in a virtual new segment is defined as a change in the objective function after modifying the beamlet intensity by one unit,^15^, ^31^ and is computed using index dose:

(8) $$\begin{array}{lll} \text{cos}t_{b} & = & \sum_{v\in b}\omega_{v}({\text{dose}}_{v}-{\text{dose}}_{v}^{p})\partial {\text{dose}}_{v}^{b}\\ & = & \sum_{v\in b}\omega_{v}(i_{v}-1){\text{dose}}_{v}^{p}\partial {\text{dose}}_{v}^{b} \end{array}$$

where $\partial {\text{dose}}_{v}^{b}$ is the dose at the voxel v deposited by the beamlet *b* with one monitoring unit (MU). Once the costs for all the virtual beamlets are computed, a new segment shape is formed at the planner's request. The sum of the price cost from all the enclosed beamlets should be the most negative possible. For MLC leaf motion constraints, the most negative sum cost is chosen using a network flow formulation.^(32)^

## III. RESULTS & DISCUSSION

**Figure 5:**
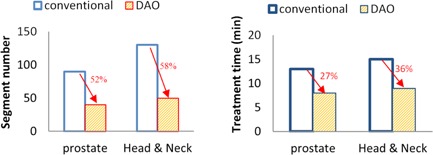
Compared to the conventional two‐step IMRT optimization, DAO reduced the treatment time using fewer segment without degrading plan quality.

For IMRT plans, DAO can effectively reduce the number of segments without degrading plan quality. This minimizes the time for patient movement and, thus, should improve treatment quality. This reduction also improves the clinical workflow and decreases wear and tear on the treatment machine. For the Siemens linacs (Siemens AG, Munich, Germany) used in our clinic, a comparison between DAO and the conventional two‐step approach was carried on 40 IMRT patients recruited in 07/2011 ~ 09/2011. Using the same beam configurations, we verified that the plans met the same optimal quality by inspecting the isodose line distributions and DVHs. All the plans were also checked against the clinical customized goals approved by the physicians. Without sacrificing the dosimetric quality, the average number of segments was reduced from 90 to 40 for the prostate cases, and from 130 to 50 for the head and neck cases, shown in Fig. 5. The dose delivery time was reduced from 13 minutes to 8 minutes for the prostate, and from 15 minutes to 9 minutes for the head and neck. This reduction in treatment time agrees with other studies: DMPO,^(13)^ half pencil beam step size for postplan optimization.^(33)^ and trust region‐like method to optimize leaf positions^(34)^ where local leaf positions were optimized exploiting gradient information of a GOF.

Back‐projection is used in CT reconstruction because it is fast and simple; index‐based DAO is also fast and simple for optimization. A simple algorithm is also good to implement because it can be checked for errors more easily than a complex one. Compare this, for instance, to conjugated gradient or quasi‐newton methods, which need to compute both the 1st and simplified 2nd order gradients to find a direction to move, and then use a golden section or similar method to determine a distance to move. Index‐dose approach is simpler in mathematics and programming. Once optimization parameters, such as gantry angles and DVH constraints, have been specified by the planner, the index‐dose optimization process can finish in single digit minutes using a Linux PC (Lenovo ThinkStation S20; Lenova Group Ltd., Beijing, China). Index‐dose optimization can also be configured to run in parallel if computing speed is a concern.

**Figure 6:**
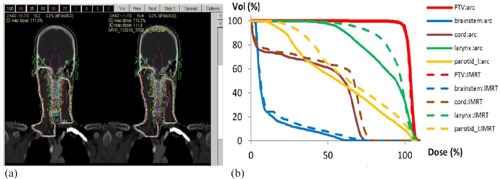
Results of rotational IMRT (arc) using index‐dose optimization: (a) coronal view of the head and neck case with isodose lines from rIMRT plan (left) and a nine‐field static IMRT plan (right); (b) DVH curves (solid line) for the rIMRT plan and the IMRT plan (dash line).

Arc therapy enables plans of comparable quality to IMRT plan to be delivered in much less time. Recently, a rotational IMRT (rIMRT) using burst mode was introduced that delivers step‐and‐shoot in a rotational manner. It turns off the beam during MLC motion.^35^, ^36^ Using index‐dose optimization, a head and neck case for rIMRT plan was retrospectively studied using the proposed DAO. The plan was not delivered since the current accelerators in our clinic are not equipped with arc features. Figure 6(a) shows a coronal view of the patient, together with isodose lines from the rIMRT plan and a nine‐beam static IMRT plan that was actually used for treatment in the clinic. Figure 6(b) depicts its DVH curves (solid line, rIMRT), compared to the nine‐beam static IMRT plan (dotted line). Better sparing for the OARs is observed for the rIMRT plan. The rIMRT plan is modeled upon a Siemens Artiste linac with a flat 6 MV beam profile. Its delivery time is estimated at around five minutes with 528 MU total. It is composed of two arcs with 39 optimization points, and each optimization point is separated at least 10° apart. The computational time for the rIMRT planning is less than 10 minutes. One challenge of rIMRT planning is to minimize its optimization points, which define segment shapes and gantry positions. This is needed to ensure smooth delivery, and allow communication and verification for the hardware controllers.

## IV. CONCLUSIONS

We have demonstrated a new index‐based DAO technique using an inverse form of back‐projection approach. The method effectively reduces treatment time by reducing the number of segments needed for step‐and‐shoot IMRT plans, and it also laid the ground work for improving the rotational IMRT with burst mode. For research purpose, the source code of PLanUNC is accessible upon request (http://planunc.radonc.unc.edu/).

## ACKNOWLEDGMENTS

The authors would like to thank Dr. A. Siochi (University of Iowa) for sharing the program for leaf sequencing, and Dr. S. K. Das (Duke University) and Dr. C. Men (Elekta Oncology) for valuable discussions on IMRT optimization. They would also like to thank the anonymous reviewers for their helpful and constructive comments that greatly contributed to improving the final version of the paper. Finally, they would like to thank the Editors for their generous comments and support during the review process.

## Supporting information

Supplementary Material

Supplementary Material
