# Supplementary figures and images for: Direct aperture optimization using an inverse form of back‐projection
Source: J Appl Clin Med Phys. 2014 Mar 6;15(2):50–9. doi: 10.1120/jacmp.v15i2.4545 (PMC5875482; doi:10.1120/jacmp.v15i2.4545)

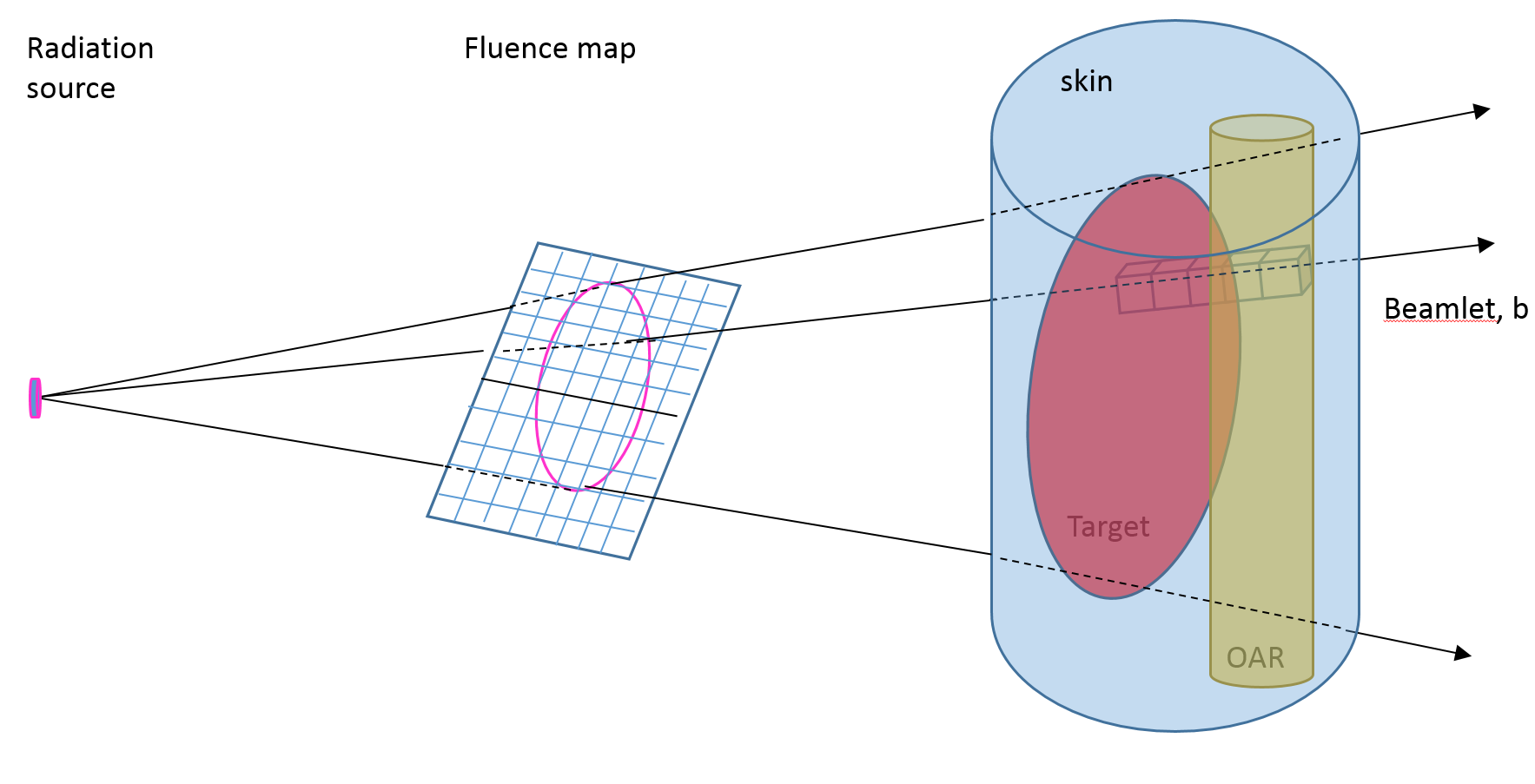

Supplement: Supplementary file 1 — Supplementary Material [file ACM2-15-50-s001.png]
